# Comprehensive profiling of the vaginal microbiome in HIV positive women using massive parallel semiconductor sequencing

**DOI:** 10.1038/srep04398

**Published:** 2014-03-18

**Authors:** Adam Ameur, Tracy L. Meiring, Ignas Bunikis, Susana Häggqvist, Cecilia Lindau, Julia Hedlund Lindberg, Inger Gustavsson, Zizipho Z. A. Mbulawa, Anna-Lise Williamson, Ulf Gyllensten

**Affiliations:** 1 Department of Immunology, Genetics and Pathology, Science for Life Laboratory Uppsala, Uppsala University, Sweden; 2 Institute of Infectious Disease and Molecular Medicine and Division of Medical Virology, Faculty of Health Sciences, University of Cape Town, South Africa; 3 National Health Laboratory Service, South Africa; 4 These authors contributed equally to this work.

## Abstract

Infections by HIV increase the risk of acquiring secondary viral and bacterial infections and methods are needed to determine the spectrum of co-infections for proper treatment. We used rolling circle amplification (RCA) and Ion Proton sequencing to investigate the vaginal microbiome of 20 HIV positive women from South Africa. A total of 46 different human papillomavirus (HPV) types were found, many of which are not detected by existing genotyping assays. Moreover, the complete genomes of two novel HPV types were determined. Abundance of HPV infections was highly correlated with real-time PCR estimates, indicating that the RCA-Proton method can be used for quantification of individual pathogens. We also identified a large number of other viral, bacterial and parasitic co-infections and the spectrum of these co-infections varied widely between individuals. Our method provides rapid detection of a broad range of pathogens and the ability to reconstruct complete genomes of novel infectious agents.

Massively parallel sequencing (MPS) provides an unprecedented opportunity to detect known viral and bacterial pathogens and identify novel infectious agents. The throughput of MPS makes it possible to study also highly complex human microbiota, such as that of the vaginal environment. The complexity of the vaginal environment is due both to the hormonal cycles, and resulting changes of the mucosa, and the multitude of sexually transmitted pathogens. Infections that compromise immune system function, such as with human immunodeficiency virus (HIV), have a strong effect on the vaginal microbiota. HIV infection increases the risk for opportunistic infections by bacteria, viruses, fungi and parasites and the development of several types of cancers (Kaposi's sarcoma, Burkitt's lymphoma, primary central nervous system lymphoma, cervical cancer) and recurring respiratory tract infections. Antiretroviral (ARV) treatment can restore the CD4+ count, but HIV infected individuals still have an increased risk of acquiring secondary infections. For patients with a reduction of immune system function, information on the viral, bacterial and parasitic co-infections may be critical for proper clinical management and choice of therapy.

MPS has been employed for genotyping of HPV in cervical cell samples by sequencing of amplicons^1^,^2^,^3^,^4^, genomic DNA from condylomas^5^ and cervical specimens^6^. Other studies have searched more broadly for infectious agents in vaginal samples using 16S rRNA gene amplicon sequencing^7^,^8^. In a recent study of the DNA in plasma, HIV positive patients showed the presence of both viral and bacterial infections not present in healthy individuals^9^. However, no studies have used the latest MPS technology for characterization of the vaginal microbiota of HIV infected women.

MPS technologies differ substantially in sequence read length, turn-around time from sample preparation to sequence generation, and cost. For the clinical usefulness of MPS, the cost per sample and the turn-around time are particularly important. The massively parallel semiconductor sequencing of Ion Proton is amendable to automation in preparation of sequencing libraries and has rapid sequence generation, making it suitable for analysis of clinical samples^10^. We have combined sample preparation enriching for circular DNA of infectious agents using rolling circle amplification (RCA), with Ion Proton sequencing to characterize the vaginal microbiota of HIV infected women.

## Results

Clinical characteristics of the 20 women are summarized in Table 1. The PAP cytology showed a High-grade Squamous Intraepithelial Lesion (HSIL) in 3 women. Six of the women had a CD4 count below 200, and 2 of these also had HSIL. The RCA-Proton method resulted in more than 7 million sequence reads per sample (Supplementary Table S1) and these were analyzed in two steps (Supplementary Figure S1). We first focused on HPV and mapped sequence reads to the reference sequences of 143 HPV types^11^ and identified the sequences of two novel HPV types. In the second step, we used the remaining reads to identify additional viruses, bacterial and parasitic co-infections.

**Figure 1:**
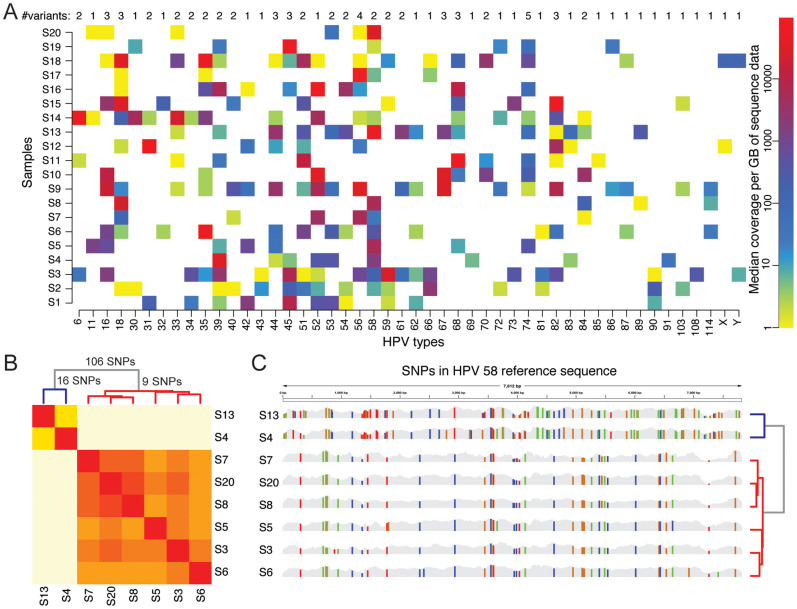
Overview of HPV types detected in the 20 HIV infected women. (A) Heat map showing all detected HPV types. Each colored square represents an HPV type (x-axis) detected in each of the samples S1–S20 (y-axis). The abundance of each infection was calculated by the median sequence coverage as detected by RCA-Proton, represented by a log-scale ranging from 1 (yellow) to 100 000 (red). Above the columns are values representing the number of distinct sequence variants detected. (B) Hierarchical clustering of SNP variation in HPV 58 for 8 individuals with high levels of HPV 58. The cluster analysis divided the samples into two distinct groups; one containing samples S4 and S19 (differing by 16 SNPs) and the other containing samples S3, S5, S6, S7 and S20 (differing by 9 SNP). The two groups differed by 106 SNPs. The large number of SNPs separating these two groups shows the presence of two distinct variants of HPV 58. (C) SNP patterns across the entire HPV 58 reference sequence for each of the samples in panel B). SNP positions are marked by vertical lines with different colors depending on the substituted nucleotide (A = green, C = blue, G = orange, T = red). The two sequence variants of HPV 58 have very distinct SNP patterns.

A total of 46 known HPV types were detected in the 20 women, with 5 to 21 types per woman (Figure 1A), which is substantially higher than the 36 types identified in commercial kits. The sequence coverage of HPV types varied by a factor of 10^5^. This may indicate large differences in HPV titer between types. Across the 20 samples the most highly abundant type was HPV 58, followed by a number of other high-risk HPVs (Supplementary Figure S2). Extensive single nucleotide polymorphism (SNP) variation was found within an HPV type between samples, consistent with the presence of different sequence variants. A cluster analysis performed for each HPV type resulted in the identification of multiple sequence variants for many of the HPVs (Figure 1A). For example, for HPV 58, which was detected in 12 of the 20 women, the cluster analysis revealed two distinct groups of HPV 58, that can be distinguished from the aligned sequences (Figure 1B, C). This extensive sequence variation within HPV types is not detected by conventional genotyping assays and might be of clinical importance.

**Figure 2:**
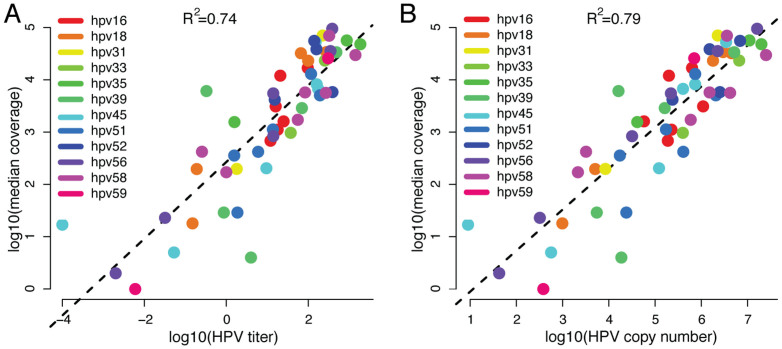
Correlation of HPV amounts estimated using real-time PCR versus RCA-Proton. (A) Comparison of HPV titer values (HPV copies per cellular equivalent, x-axis) from real-time PCR versus median sequence coverage obtained by RCA-Proton (y-axis). (B) Correlation of HPV copy number (x-axis) versus RCA-Proton (y-axis).

The sequence-based identification of HPV types was compared to genotyping using the Roche Linear Array HPV Genotyping Test and the *HPVIR* assay. RCA-Proton identified 96% (108/112) of HPV infections detected by genotyping (Table 1). However, the genotyping assays detected only 49% (108/222) of the HPVs identified by sequencing. For the remaining 51% (114 observations), 61 were HPV types not included in the genotyping assays and 53 were missed because of other reasons, such as SNP/indel variation within the PCR primer sequences used for genotyping. To determine if the RCA-Proton sequence coverage reflected the abundance of an HPV type, we compared the sequence coverage with the data from the *HPVIR* real-time PCR. The sequence coverage was highly correlated with both total viral amount (HPV copy number, R^2^ = 0.79) and viral titer (HPV copy number per cellular equivalent, R^2^ = 0.73) (Figure 2). Thus, the RCA-Proton sequence coverage reliably estimates abundance of individual HPV types, even when a sample contains multiple HPV types.

**Figure 3:**
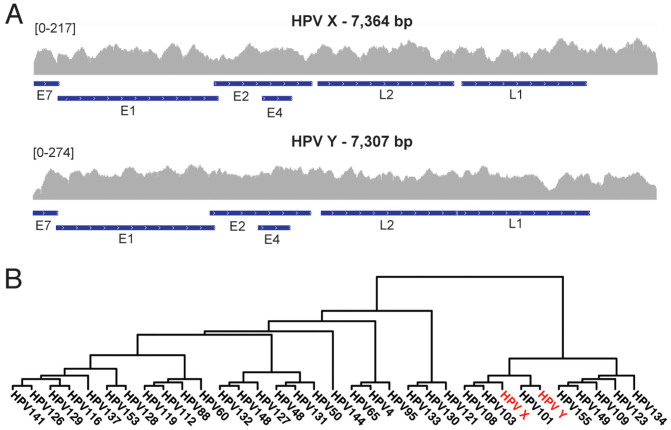
Classification of novel HPV types. (A) Coverage and profile of reads from sample S18 mapped to the assembled reference sequences for HPV X (top) and HPV Y (bottom). The location of genes is indicated by blue lines below the coverage plots. (B) Dendrogram based on the L1 gene of HPV X and Y together with reference sequences from the HPV gamma branch. The tree shows that HPV 101, 103 and 108 are the evolutionary closest relatives of HPV X and Y. The sub-tree containing HPVs X, Y, 101, 103 and 108 had a bootstrap value of 100, meaning that it was present in 100% of re-samplings.

To search for novel HPV types, not present among the 143 HPV reference genomes, we removed all reads mapping to known HPVs or to the human genome and performed a *de novo* assembly of remaining reads. This resulted in the identification of two novel HPV types, denoted HPV X and Y, and an assembly of their complete genome sequences (Figure 3A). One of the women (S18) was infected with both of these novel HPVs. Additionally, HPV Y was detected in S3 and HPV X was found at low levels in S12. In order for a previously unknown HPV type to be classified as novel, the L1 gene sequence should show at most 90% similarity to any previous described type in the database^12^. A cluster analysis based on the L1 gene sequence showed that both HPV X and Y were most closely related to HPV 101, 103 and 108 in the HPV-gamma group (Figure 3B). HPV X and Y showed 78% and 67% similarity in the L1 gene to their closest relative, respectively and both therefore satisfy the criteria for classification as novel HPV types (see Supplementary Table S2).

**Figure 4:**
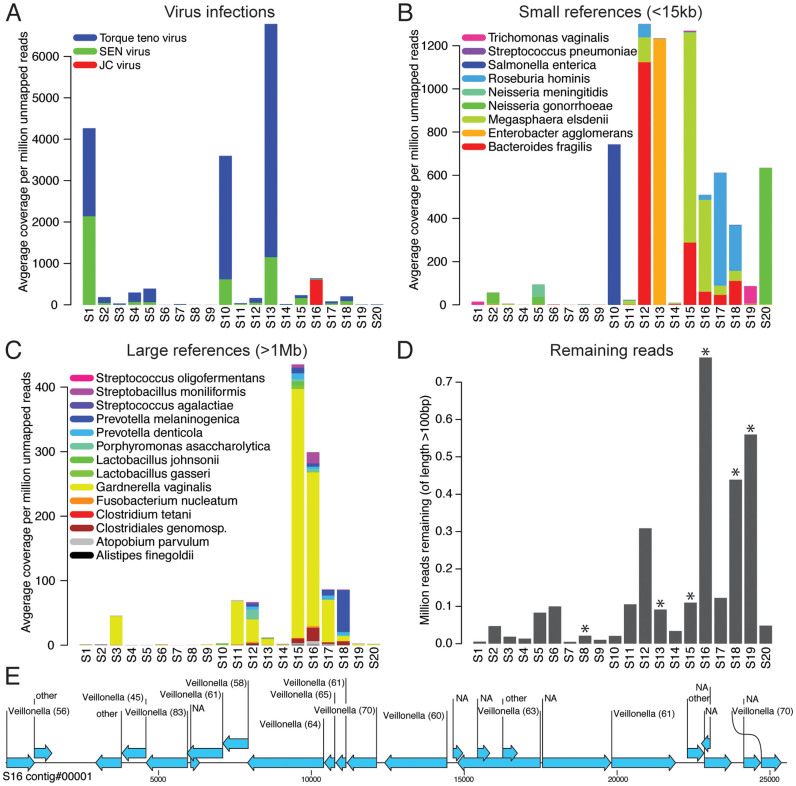
Distribution of viruses, bacteria and parasite sequences in the study women. (A) The colored bars represent the average sequence coverage over viral references per million reads, for reads that could not be mapped to any HPV reference or to the human genome (average coverage per million unmapped reads). (B) Average coverage per million unmapped reads for smaller, non-viral, sequences of length < 15 kb). These sequences originate from bacterial sequences (plasmids, transposons etc.) or from genes found in parasites. (C) Average coverage per million unmapped reads for complete bacterial genomes with lengths > 1 Mb. (D) Number of long reads (100 bp or more) remaining after having removed those mapping to the human genome, HPV reference sequences or the additional infections in panels A–C. Since quality trimming was performed during Ion Proton sequencing, these reads have high quality across the entire read length. Asterisks above the bars are used to mark samples where long contigs of unknown origin could be assembled (see supplementary Table S4). (E) Open reading frames (ORFs) within a ~25 kb long contig resulting from *de novo* assembly of the remaining reads from sample S16. The arrows indicate the position and direction of transcription for the ORFs. Numbers in parenthesis show the translated ORF sequence similarity to *Veillonella* proteins as detected by blastx.

Through a strategy of *de novo* assembly of sequence reads not mapping to the human genome or to any HPV type, we found a series of additional (non-HPV) viruses, chromosomal and extrachromosomal elements (plasmids and transposons) from pathogenic, potentially pathogenic and non-pathogenic bacteria, as well as parasites in the samples (Supplementary Table S3). Among additional viruses detected were *Torque teno* and *SEN* virus, found in multiple samples, and the *JC* virus present only in one individual (Figure 4A). Short reference sequences from bacteria or parasites were analyzed separately and within this category a large variation was found between women with some species detected in multiple samples (e.g. *Neisseria gonorrhoeae* and *Bacteroides fragilis*) and others occurring in only a single sample (e.g. *Trichomonas vaginalis* and *Salmonella enterica*) (Figure 4B). We further identified sequences originating from large circular bacterial genomes of lengths 1 Mb or more, and within this category we identified a broad range of infections with *Gardnerella vaginalis* being the most abundant (Figure 4C). As seen in supplementary Figure S3, there is a high and uniform sequence read coverage across the reference sequences for several infectious agents. This implies that the RCA-Proton data can be used also to study genetic variation for bacterial and viral (non-HPV) infections.

For some samples, there remained a substantial number of long and high-quality sequence reads after the analysis of non-HPV infections (see Figure 4D). These reads did not match any of the infections analyzed above and therefore they may represent additional infectious agents for which the DNA sequences are still unknown. In six of the samples, we were able to assemble a total of 62 long contigs (of lengths 4 kb to over 25 kb) with no nucleotide sequence similarity to any known organism (data not shown). However, all of the assembled contigs contained a large number of open reading frames (ORFs) suggesting that they originate from DNA molecules densely packed with protein coding genes (see Figure 4E). By a protein similarity search within the ORFs we could conclude that all six samples contained sequences showing resemblance to protein-coding regions from various bacterial species (Supplementary Table S4), with *Clostridium* being the most common match among the women. Sample S16 showed the highest abundance of unknown bacterial sequences. The majority of contigs in S16 contained ORFs that showed resemblance to *Clostridium*, while others were assigned to other species including *Veillonella*, *Lactobacillus* and many more. This demonstrates that S16 carries an infection of a bacteria with a presently undescribed genome. However, it remains unclear whether all the sequences in S16 comes from one single infection, or if there are in fact multiple infections of uncharacterized bacteria in the sample. In conclusion, our results show that the RCA-Proton method can be used not only for the analysis of known viral, bacterial and parasitic infections, but also for the genome assembly and initial classification of previously uncharacterized infectious organisms.

## Discussion

The RCA-Proton method enabled us to study the infection burden in HIV positive women at a new level of resolution. The prevalence of HPV is known to be substantially higher in HIV infected individuals^13^, but previous studies have been limited to the range of HPV types included in the genotyping assays. Our study provides an estimate of the full range of infecting HPV types, and the results shows the presence of over 40 HPV types in these women, with a single woman carrying as many as 21 HPV types. In a previous MPS study we examined a single HIV positive woman, and similarly detected multiple HPV types, some not included in the genotyping assays^6^. Our finding of two novel HPV types based on the analysis of only 20 individuals implies that multiple HPV types likely remain to be discovered and may contribute to tumor development. The oncogenic potential of many HPV types not included in the genotyping assays is presently unknown, but since HIV positive women have a less efficient immune response to co-infections such as HPV, they may also be less able to handle the transformation process initiated by otherwise less oncogenic HPV types. HPV types classified as low risk in otherwise healthy women may therefore contribute to tumor development when the immune system is not functioning properly.

The RCA-Proton assay resulted in a broad screening of the infectious burden of these women and identified a large number of DNA molecules from co-infecting bacterial plasmids, viruses and parasite genomes. Except HPV, infections by *Torque teno*, SEN-V and *JC* viruses were detected. *Torque teno* is asymptomatic, SEN-V associated with post-transfusion hepatitis, and the *JC* virus asymptomatic but may be activated in immunosuppressed individuals and result in damage to the brain and kidneys. Among the bacterial infections are human pathogens such as *Streptococcus pneumoniae*, *Neisseria meningitides* and *Neisseria gonorrhoeae*. *Enterobacter agglomerans* is an opportunistic pathogen and cause wound, blood, and urinary tract infections in immunosuppressed individuals, and *Bacteroides fragilis* is involved in 90% of anaerobic peritoneal infections. *Streptococcus agalactiae* is found in the gastrointestinal flora of humans and if transferred to a neonate can cause serious streptococcal infection (bacterial septicemia of the newborn). G*ardnerella vaginalis* can cause bacterial vaginosis in some women. We also identified the parasite *Trichomonas vaginalis*, which is a flagellated protozoan causing trichomoniasis.

Our results also showed the presence of additional infectious agents, for which the genome sequences have not been determined. In several samples it was possible to reconstruct large pieces of DNA that did not match any known reference sequence. The analyses of protein-coding domains revealed that many of these sequences represent additional, previously uncharacterized, bacterial infections. The most common infection showed 50–75% amino acid similarity to *Clostridium*, a genus of Gram-positive bacteria that include species of common free-living bacteria as well as important pathogens. It should be noted there are many uncertainties in the prediction of the closest related organism for these unknown DNA sequences, and these results should be seen as a preliminary rather than a final classification. Although further bioinformatics and biomedical analyses are required to confirm the genomic origins of these sequences and elucidate the role of these previously unknown infections in human disease, our results shows that the RCA-Proton method can be used as a first step in identifying novel pathogens.

The RCA based sequencing has many benefits but there are also some limitations with this approach. One of the main limiting factors is that RCA requires deeper sequencing as compared to 16S rRNA sequencing for metagenomic sequencing of bacterial communities. This is because the RCA method amplifies larger parts of genomes instead of just a single amplicon, and thus requires more reads to identify the bacterial species present in a sample. Moreover, the RCA reaction generates a relatively high proportion of reads originating from the host genome, thereby increasing the need for deep sequencing even further. When it comes to analyses of HPVs, the RCA method is not suitable for studying HPV integration into the human genome. Since small circular genomes are preferentially amplified in the RCA reaction the integrated HPVs will typically not be sequenced at high coverage.

We have shown that the RCA-Proton method can be used for comprehensive profiling of the infection burden in vaginal samples. Since many bacteria and viruses are difficult or impossible to isolate and grow in aerobic conditions or outside their host or community, *in vitro* genetic screening is the most powerful approach to assess the microbiota. For MPS to be suitable for such analyses it has to be economically feasible to use. By barcoding of samples prior to Ion Proton sequencing, the price tag per sample can be reduced substantially. Further increases of the sequence throughput, such as that predicted for the Ion Proton chip PII, and the use of a higher number of barcodes, would make it possible to analyze as many as 100 samples in a single run, dramatically reducing the cost per sample. The run time on the Ion Proton instrument is only a few hours, and it is therefore possible to complete the process from sample preparation to generation of medically actionable data, in a few days. In summary, application of the latest MPS technology, such as the RCA-Proton method employed here, is likely to reveal a number of known and novel co-infections occurring in many infectious diseases. The pattern of co-infections in the present study varied dramatically between women, with a unique spectrum of viral, bacterial and parasitic co-infections present in each sample. Further studies of the co-infection profile will be important in order to determine their importance for proper clinical management of individual patients.

## Methods

### Study population and specimens

The cervical samples are from a study conducted at the Empilisweni centre, Gugulethu, Cape Town, South Africa, conducted from 2006 to 2009. Study participants were recruited to Manyanani clinic with their sexual partner. The Research Ethics Committee of the University of Cape Town approved all aspects of the investigation (reference: 258/2006) and informed consent was obtained. All experiments were carried out in accordance with the approved guidelines. Samples were collected and stored as described^13^,^14^,^15^,^16^. DNA was extracted from cervical cells using the MagNA Pure Compact Nucleic Acid Isolation Kit (Roche diagnostics, Mannheim, Germany) and automated Roche MagNA Pure Compact machine. DNA was stored at −20°C. The sub study described in this paper is on genital samples from 20 HIV positive women.

### Detection and quantification of HPV DNA

The samples were HPV typed using two different systems. Samples S1–S6 and S8–S20 were typed using the Roche Linear Array HPV Genotyping Test. This line-blot assay individually identifies 36 HPV genotypes (see Table 1). All samples were also analyzed using the *HPVIR* real-time PCR assay as described^17^. This assay is based on four parallel real-time PCRs from each DNA sample, one reaction to quantify the amount of a human single-copy gene (house-keeping gene; Homo sapiens hydroxymethylbilane synthase (*HMBS*); GenBank accession no. M95623.1) and the three other reactions to detect and quantify HPV 16, 18, 31, 33, 35, 39, 45, 51, 52, 56, 58, and 59. The threshold for a positive HPV type was set at 10 copies per PCR. Similarly, as a threshold for inclusion in the study a copy number of 10 genomic equivalents were used. HPV copies per cell (titer) were calculated by dividing HPV copies per sample by copies of *HMBS*.

### Multiply-primed RCA

We used a version of the rolling circle amplification (RCA) method designed to more specifically target human papillomavirus (HPV)^18^. Although this primer set has a higher specificity for HPV than the random hexamer primers in the commercial RCA kit, it does not exclude amplification of other circular DNA molecules. The vaginal DNA sample (3 μl) and primers were denatured at 95°C for 3 minutes, cooled to 4°C and placed on ice. The denatured sample was then added to 10 μl of the amplification solution, with the 23 specific primers at a final concentration of 0·4 μM each, 1X phi29 DNA polymerase buffer, 2 ng/μl bovine serum albumin (BSA), 15 mM dNTPs, 2 U/μl of phi29 DNA polymerase (Thermo Scientific, Fermentas, Sweden) and dH_2_O in a total volume of 50 μl, as described^18^. The sample was incubated at 30°C for 18 hours, followed by inactivation of the enzyme at 65°C for 10 minutes, and then stored at −20°C.

### Massively parallel DNA sequencing using the Ion Proton technology

Sequencing of RCA products was performed using the Ion Proton Sequencer (Life Technologies). A total of 500 ng of each sample was fragmented using the Covaris S2 system with a target peak of 150 bp. End repair and adaptor ligation were performed by the AB Library Builder System (Life Technologies). The samples were amplified, purified with Agencourt AMPure XP beads (Beckman Coulter Genomics) and size selected with a target peak of 220 bp (Blue Pippin, Sage Science). Library size and concentration were assessed on a Bioanalyzer High Sensitivity Chip (Agilent Technologies) and the samples were diluted to a concentration of 12 pM. The 20 samples were barcoded and pooled together in four sets of five samples each before template preparation with the Ion PI Template OT2 200 kit on the OneTouch 2 instrument (Life Technologies). The templated Ion Spheres for each pool of five samples were loaded onto a PI.v1 chip and sequenced on the Ion Proton using the Ion PI Sequencing 200 kit (400 flows). Quality trimming was performed to ensure high quality throughout the whole reads, using the default settings in Torrent Suite v3.4.2. The quality trimming removes low-quality base calls at the 3′ end of the reads.

### Statistical and bioinformatics analysis of HPV infections

Our analysis pipeline is based on successive rounds of mapping and subtraction of mapped reads (supplementary Figure S1). First, all Ion Proton reads were mapped against a reference panel consisting of the human genome sequence (hg19 assembly) and 143 available HPV sequences from the PaVE database (http://pave.niaid.nih.gov/)^11^. The mapping was performed with the TMAP alignment plug-in in Torrent Suite v3.4.2. Reads not mapping to any known HPV type or the human reference genome were extracted and used for *de novo* assembly using Newbler v2.6. Among the assembled sequence contigs, we screened for novel HPV genomes by applying two filtering criteria: *i*) a contig length close to those of known HPV-types, i.e. between 7000 and 9000 bp and *ii*) a blastx search should identify the HPV proteins L1, L2, E1 and E2. Novel HPV genomes detected using this approach were added to the references sequence after which the process was repeated until no additional HPV types were detected. This resulted in the identification of two novel HPV types, denoted HPV X and HPV Y.

### Quantification of HPV types and identification of HPV subtypes

The abundance of each HPV type was estimated from the Ion Proton reads by measuring the median coverage over the HPV references. Single nucleotide polymorphism (SNP) calling was performed by the Variant Caller in Torrent Suite v3.4.2, and the resulting SNP information was used to determine sequence variation for each HPV. Only those HPV types detected in samples with sufficiently high read depth were used for this analysis (90% of the bases were required to have ≥10× coverage). For each HPV type and sample satisfying the coverage criteria, all homozygous SNP calls were extracted. A distance matrix was computed for each HPV type based on the number of SNP differences between samples infected by the same HPV. The distance matrixes were used as a basis for a clustering analysis (using custom R scripts) to obtain number of distinct variants for each HPV. A distance of at least 30 SNPs was required for two HPVs belonging to the same type to be classed as different sequence variants.

### Detection of non-HPV co-infections with available reference genomes

To search for additional infectious agents, we performed a *de novo* assembly (using Newbler) of all reads not mapped to any HPV (including HPV X and Y) or the human reference genome (hg19) for each of the samples (Table 1). All assembled contigs of length > 1 kb were then matched against the NCBI nucleotide database (using blastn) and all hits having at least 95% similarity were recorded. The complete reference sequences for these hits represent various non-HPV infectious agents (viruses, plasmids, bacteria etc). The complete sequences for all of these infectious agents were downloaded and Ion Proton reads were mapped back to these reference sequences to obtain measurements for all identified infections in all of the samples.

### Detection of uncharacterized infectious organisms with no available reference

Reads that could not be mapped to the human reference, to HPVs or to other co-infections were used to search for additional infections with unknown reference genomes. A *de novo* assembly (using Newbler) resulted in the detection of 62 long contigs (of length at least 4 kb) in six of the samples, which could not be matched to the NCBI nucleotide sequence database using blastn. Predicted open reading frames (ORFs) encoding proteins of at least 100 amino acids (aa) were extracted from the contigs using CLCbio. The predicted ORF sequences were matched with blastx to identify conserved protein domains. For each contig, the organism showing the highest total number of ORF hits was considered to be the evolutionarily closest species.

## Author Contributions

A.A., T.M., Z.M., A.L.W. and U.G. designed the study. T.M., S.H., C.L., J.H.L. and I.G. developed the workflow for sample preparation and sequencing. A.A., I.B. and T.M. performed the data analysis and results were interpreted together with A.L.W. and U.G. A.A. and U.G. wrote the main manuscript text. All authors reviewed the manuscript.

## Supplementary Material

Supplementary Information Supplementary information

## Figures and Tables

**Table 1:** Patient characteristics and HPV typing results

Sample #	Age	Cytology^b^	CD4	HPV genotyping^a^	HPV typing by RCA-Proton
S1	25	Ascus	102	31, 42, 45, 52, 53, 61, 62	31, 34, 39, 42, 45, 52, 53, 54, 59, 62, 90
S2	20	LSIL	473	18, 45, 51, 54, 58, 66, 72, 81	18, 30, 39, 40, 43, 45, 51, 52, 54, 58, 59, 66, 72, 81, 90, 103
S3	18	LSIL	244	6, 16, 39, 45, 56, 58, 59, 61, 62, 66, 73, 81, 89	6, 16, 34, 35, 39, 43, 45, 51, 52, 56, 58, 59, 61, 62, 66, 73, 81, 82, 90, 108
S4	25	Normal	188	39, 45, 53, 58, 83	39, 44, 45, 52, 53, 58, 69, 83, 91
S5	30	LSIL	71	11, 16, 42, 58,	11, 16, 39, 42, 44, 58, 68, 74
S6	35	HSIL	480	16, 35, 44, 51, 53, 58, 62, 68, 81, 84	16, 18, 32, 35, 44, 51, 53, 54, 58, 62, 81, 82, 87, 114
S7	22	LSIL	285	52, 56	18, 40, 52, 56, 58, 84
S8	32	HSIL	120	18, 58, 84, 89	18, 58, 84, 89, 114
S9	28	LSIL	330	16, 18, 44, 52, 53, 56, 61, 62, 72, 82, 84	16, 18, 33, 35, 40, 42, 44, 51, 52, 53, 56, 61, 62, 67, 72, 74, 82, 86, 87, 103, 114
S10	23	Normal	308	16, 52, 70, 84	16, 35, 52, 67, 68, 70, 74, 84
S11	19	LSIL	302	39, 51, 68	6, 33, 39, 51, 68, 70, 74, 82, 85
S12	38	HSIL	56	31, 56, 82, 83	18, 31, 42, 44, 51, 56, 82, 83
S13	36	Normal	250	51, 53, 58, 61, 83, 89	33, 39, 44, 51, 53, 54, 58, 61, 62, 67, 68, 74, 82, 83, 84, 89
S14	33	LSIL	254	6, 18, 33, 35, 52, 56, 72, 82	6, 11, 18, 30, 31, 33, 34, 35, 44, 52, 56, 58, 68, 72, 74, 82, 84
S15	32	LSIL	144	16, 18, 51, 59, 73, 82	16, 18, 32, 40, 51, 59, 68, 73, 82, 103
S16	20	LSIL	465	35, 39, 45, 52, 54	18, 35, 39, 42, 45, 52, 54, 56, 68, 74
S17	49	LSIL	449	56, 66	18, 35, 56, 58, 66
S18	32	LSIL	225	18, 33, 35, 51, 68, 70	16, 18, 33, 35, 39, 44, 45, 51, 52, 56, 59, 66, 68, 70, 74, 87
S19	49	LSIL	677	45, 72	30, 39, 45, 58, 72, 74, 86
S20	38	LSIL	384	53, 58	11, 16, 33, 53, 56, 58

^a^The HPV typing for samples S1–S6, S8–S20 were performed using the Roche HPV genotyping Linear Array which detects the HPV types 6, 11, 16, 18, 26, 31, 33, 34, 35, 39, 40, 42, 44, 45, 51, 52, 53, 54, 56, 58, 59, 61, 62, 66, 67, 68, 69, 70, 71, 72, 73, 81, 82, 83, 84 and 89. All samples were also HPV typed using the HPVIR assay which detects and quantifies the HPV types 16, 18, 31, 33, 35, 39, 45, 51, 52, 56, 58 and 59. Number in bold in the HPV genotyping column are types not detected by the sequence-based analysis, and numbers in bold in the RCA-Proton column are types not detected in the genotyping.

^b^PAP smear cytology. HSIL; High-grade Squamous Intraepithelial Lesion, LSIL; Low-grade Squamous Intraepithelial Lesion, Ascus; atypical cells of undetermined significance.
