# Supplementary material for: Comprehensive profiling of the vaginal microbiome in HIV positive women using massive parallel semiconductor sequencing
Source: Sci Rep. 2014 Mar 18;4:4398. doi: 10.1038/srep04398 (PMC3957130; doi:10.1038/srep04398)
Supplement: Supplementary Information [file srep04398-s1.pdf]

Supplementary Information:

Comprehensive profiling of the vaginal microbiome in HIV positive women using massive parallel semiconductor sequencing

Adam Ameur *[a], Tracy L. Meiring *[b], Ignas Bunikis [a], Susana Häggqvist [a], Cecilia Lindau [a], Julia Hedlund Lindberg [a], Inger Gustavsson [a], Zizipho Z.A. Mbulawa [b,c], Anna-Lise Williamson# [b,c], Ulf Gyllensten #[a]

[a] Department of Immunology, Genetics and Pathology, Science for Life Laboratory Uppsala, Uppsala University, Sweden

[b] Institute of Infectious Disease and Molecular Medicine and Division of Medical Virology, Faculty of Health Sciences, University of Cape Town, South Africa

[c] National Health Laboratory Service, South Africa

Supplementary Figures

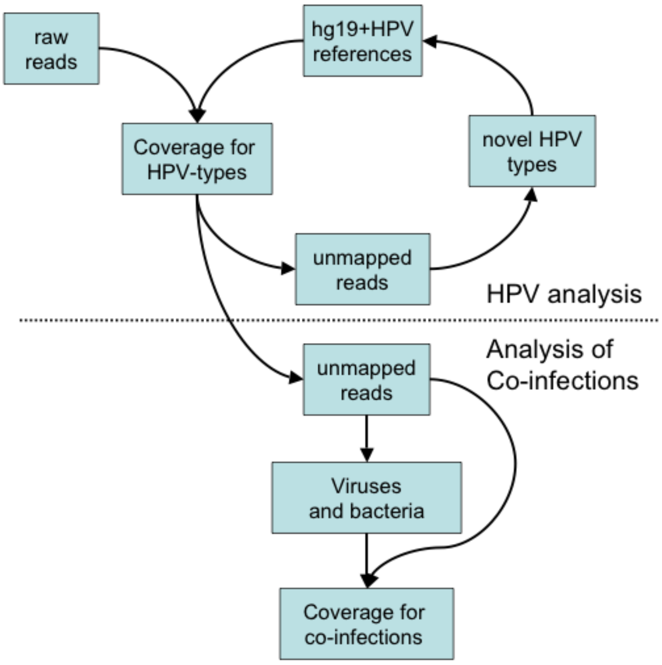

Supplementary Figure S1. Overview of bioinformatics analysis strategy. HPV infections are detected through an iterative process. First, sequence reads are mapped to a combined reference consisting of the entire human genome sequence as well as references for known HPV types, resulting in a coverage profile for each of the HPVs. The reads which could not be aligned to any HPV or the human reference (unmapped reads) are processed by a *de novo* assembly, and the resulting contigs are screened for similarities to general HPV gene features. In this way we can detect novel HPV sequences. The novel HPVs are then added to the reference file after which a re-alignment is performed against the updated reference. This process continues until no more novel HPV types are detected. After the HPV analysis is complete, we search for additional non-HPV infections among the remaining unmapped reads. A *de novo* assembly is performed and the resulting contigs are used in BLAST searches against a database with all available nucleotide sequences. Reference sequences for viruses, bacteria and other infectious agents that show very high sequence similarity to the assembled contigs are extracted, and then the unmapped reads are re-aligned against those. In this way we obtain a sequence read coverage that can be used for quantification of the levels of various co-infections.

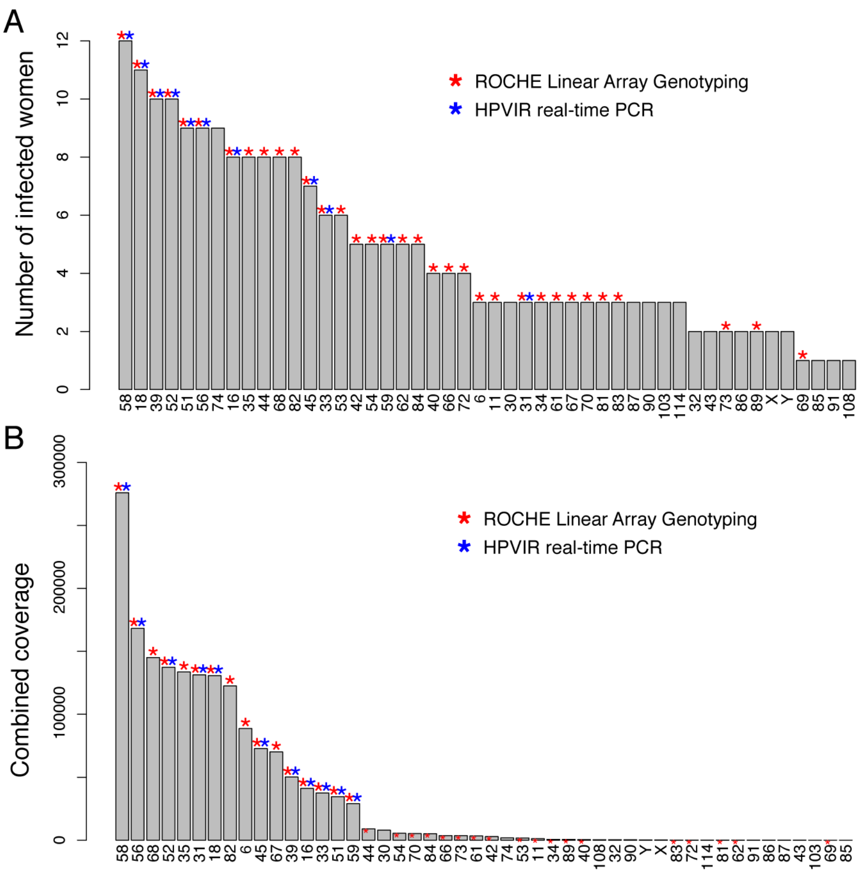

Supplementary Figure S2. Abundance of HPV types in the 20 samples. A) The gray bars show the number of infected women for each HPV type. In total 48 HPV genotypes were identified (including two novel genotypes HPV X and Y), with some being much more frequent compared to others. **B)** Combined coverage obtained for each HPV type in all of the women. All of the HPV infections with high coverage are present in the ROCHE Linear Assay Genotyping (marked by red asterisks) and most of them are also included in the HPVIR real-time PCR test (blue asterisks). There is a long tail of HPV types with low coverage, some of which are not detectable by the genotyping or real-time PCR.

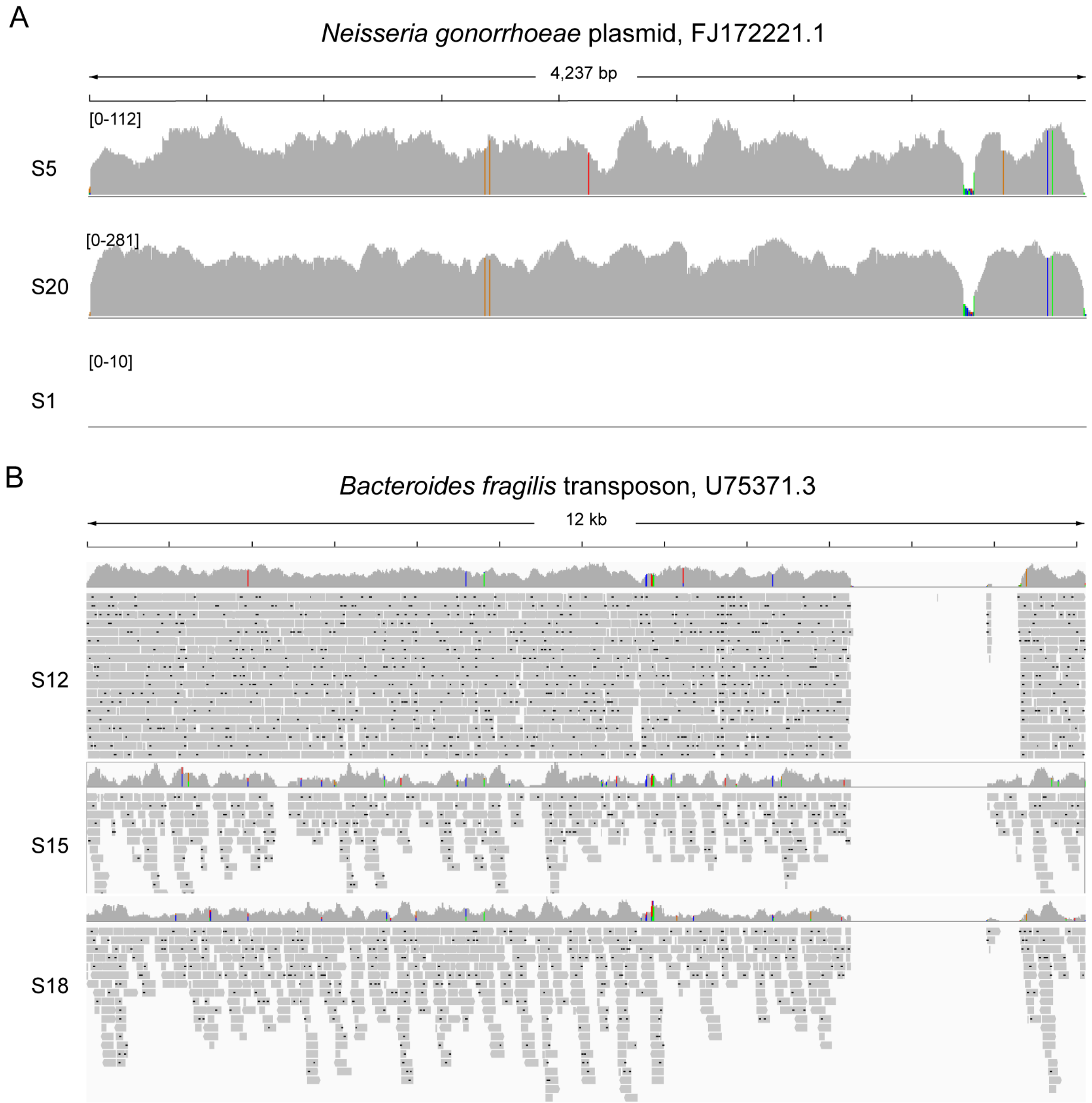

Supplementary Figure S3. Sequence coverage for non-HPV infections. A) Coverage profile for an over 4 kb piece of the *Neisseria gonorrhoeae* plasmid in three samples (S1, S5 and S20). The plasmid is clearly present in samples S5 and S20 where the coverage reaches 112X and 281X respectively. In S1 there is not a single read mapped to the plasmid reference. SNPs in samples S5 and S20 are shown as vertical colored lines. There are clearly a number of SNPs both in S5 and S20 of which many are detected in both samples. It is thus possible to study bacterial co-infections at high resolution and even to determine genetic variation between samples. **B)** Coverage profile and individual reads for the *Bacteroides fragilis* transposon U75371.3 in samples S12, S15 and S18. The samples show high coverage across the reference except for a ~ 2kb region that appears to be deleted in all three samples.

Supplementary Tables

Supplementary Table S1. Ion Proton run information for the 20 samples.

Sample #	Bases	>=Q20 Bases	No of reads	Mapped reads	Avg read length[a]
**S1**	1,123,094,246	845,921,658	9,340,619	9,293,317	120
**S2**	1,127,774,058	860,729,845	9,475,023	9,357,856	119
**S3**	1,124,994,779	866,858,532	9,355,034	9,254,266	120
**S4**	1,173,214,481	890,087,842	9,910,115	9,850,996	118
**S5**	949,846,119	726,488,153	8,116,682	7,960,205	117
**S6**	1,286,638,236	901,835,493	10,370,987	10,164,937	124
**S7**	1,325,653,574	935,382,902	10,709,737	10,613,131	123
**S8**	1,228,708,905	870,224,738	9,896,537	9,789,102	124
**S9**	891,389,357	635,020,898	7,052,038	6,967,029	126
**S10**	1,359,245,507	940,175,509	10,974,157	10,833,021	123
**S11**	1,967,847,805	1,314,580,625	17,842,951	16,985,972	110
**S12**	1,862,343,610	1,276,740,852	16,246,875	15,550,508	114
**S13**	1,857,594,885	1,243,793,198	16,507,559	16,091,184	112
**S14**	1,555,333,269	1,040,171,410	13,725,450	13,482,576	113
**S15**	1,888,085,964	1,257,753,806	16,665,679	16,240,891	113
**S16**	1,180,150,019	837,426,989	9,905,939	8,715,880	119
**S17**	1,354,079,231	954,439,784	11,522,705	11,214,934	117
**S18**	1,199,675,779	853,206,452	10,142,966	9,349,135	118
**S19**	1,056,016,718	733,479,972	8,962,491	8,131,553	117
**S20**	4,400,023,141	3,094,815,089	38,899,388	38,388,505	113

[a] Read lengths after quality trimming

Supplementary Table S2. Sequence similarity within the L1 gene. Novel HPV types (HPV X and Y) are compared to their evolutionarily closest relatives. In the upper right corner are numbers representing the percent similarity, in the bottom right triangle are number of identical nucleotides.

	HPV 101	HPV X	HPV Y	HPV 103	HPV 108
**HPV 101**		78.4	65.57	67.05	67.76
**HPV X**	1216		65.77	66.28	67.12
**HPV Y**	1021	1024		67.44	66.67
**HPV 103**	1046	1036	1052		75.24
**HPV 108**	1057	1047	1040	1167	

Supplementary Table S3. Reference sequences from viral, bacterial and parasitic co-infections.

	Name	Type	Accession	Ref length
1	***Alistipes finegoldii***	complete genome	CP003274.1	3.728 Mb
2	***Atopobium parvulum***	complete genome	CP001721.1	1.541 Mb
3	***Bacteroides fragilis***	transposon	U75371.3	12 kb
4	***Clostridiales genomosp.***	complete genome	CP001850.2	1.806 Mb
5	***Clostridium tetani***	complete genome	AE015927.1	2.794 Mb
6	***Enterobacter agglomerans***	plasmid	AF014880.1	2.492 kb
7	***Fusobacterium nucleatum***	complete genome	AE009951.2	2.171 Mb
8	***Gardnerella vaginalis***	complete genome	NC_013721.1	1.614 Mb
9	***JC virus***	complete genome	AF004349.1	5.112 kb
10	***Lactobacillus gasseri***	complete genome	CP000413.1	1.891 Mb
11	***Lactobacillus johnsonii***	complete genome	NC_005362.1	1.989 Mb
12	***Megasphaera elsdenii***	ribosomal RNA	NR_103173.1	2.907 kb
13	***Neisseria gonorrhoeae***	plasmid	FJ172221.1	6.053 kb
14	***Neisseria meningitidis***	plasmid	AF126482.1	5.589 kb
15	***Porphyromonas asaccharolytica***	complete genome	NC_015501.1	2.182 Mb
16	***Prevotella denticola***	complete genome	CP002589.1	2.932 Mb
17	***Prevotella melaninogenica***	complete genome	NC_014370.1	3.162 Mb
18	***Roseburia hominis***	ribosomal RNA	NR_076921.1	2.885 kb
19	***Salmonella enterica***	plasmid	NC_021157.1	4.668 kb
20	***SEN virus***	virus	AY183662.1	3.076 kb
21	***Streptococcus agalactiae***	complete genome	NC_021485.1	2.135 Mb
22	***Streptococcus oligofermentans***	complete genome	NC_021175.1	2.138 Mb
23	***Streptococcus pneumoniae***	transposon	KC488256.1	57 kb
24	***Trichomonas vaginalis***	hypothetical protein	XM_001319996.1	3.298 Mb
25	***Torque teno virus***	virus	AM712004.1	3.754 kb

Supplementary Table S4. Long contigs (at least 4kb) for which a blastn analysis gives no hit to any known reference sequence.

Sample	Contig#	Contig length	Contig reads	total ORFs[a]	nr ORF hits[b]	closest relative	Highest amino acid similarity
S5	00001	12179	19262	10	2	*Bacillus*	54
S8	00001	4163	461	7	3	*Eubacterium*	60
S8	00002	4298	2712	7	2	*Metascardovia*	54
S13	00001	9648	14501	10	8	*Streptococcus*	74
S13	00002	4902	527	4	3	*Lactobacillus*	95
S15	00001	6906	735	9	5	*Clostridium*	54
S15	00002	5556	582	14	6	*Ruminococcus*	55
S15	00003	4148	1145	6	2	*Streptococcus*	64
S15	00004	5321	496	10	4	*Lachnospiraceae*	60
S15	00005	4383	573	7	2	*Clostridium*	54
S15	00006	4256	441	5	3	*Rhodococcus*	54
S16	00001	25555	30141	24	13	*Veillonella*	62
S16	00010	9868	991	14	6	*Clostridium*	59
S16	00011	9716	1366	17	10	*Clostridium*	68
S16	00020	7835	1087	13	6	*Clostridium*	70
S16	00021	7613	788	12	4	*Clostridiales*	68
S16	00022	7561	931	13	7	*Clostridium*	63
S16	00024	7380	1023	19	2	*Lactobacillus*	50
S16	00025	7269	832	9	2	*Enterococcus*	41
S16	00026	6961	897	9	5	*Clostridium*	63
S16	00028	6586	649	9	5	*Clostridium*	68
S16	00029	6490	998	7	3	*Streptococcus*	66
S16	00030	6425	31051	6	4	*Prevotella*	87
S16	00032	6274	577	7	6	*Paenibacillus*	61
S16	00033	6248	722	9	4	*Clostridium*	60
S16	00036	6035	667	5	4	*Clostridium*	59
S16	00037	5982	516	11	4	*Gardnerella*	69
S16	00038	5849	853	7	4	*Eubacterium*	67
S16	00039	5834	540	7	3	*Clostridiales*	48
S16	00041	5732	604	9	6	*Lachnospiraceae*	67
S16	00042	5717	613	7	6	*Clostridium*	71
S16	00043	5632	831	6	1	*Actinomyces*	44
S16	00045	5612	690	8	5	*Oribacterium*	73
S16	00052	5269	480	12	5	*Lactobacillus*	68
S16	00056	5149	463	6	5	*Ruminococcus*	67
S16	00057	5107	586	5	4	*Streptococcus*	58
S16	00059	4981	531	5	3	*Clostridiales*	70
S16	00061	4927	427	5	4	*Firmicutes*	51
S16	00063	4881	407	6	4	*Clostridium*	58
S16	00066	4784	491	6	4	*Clostridium*	56
S16	00067	4754	566	8	5	*Clostridium*	75
S16	00068	4746	403	6	4	*Fusobacterium*	77
S16	00069	4738	580	8	2	*Parvimonas*	67
S16	00072	4656	490	7	3	*Lactobacillus*	62
S16	00075	4581	437	5	4	*Clostridium*	69
S16	00076	4502	394	7	5	*Clostridium*	58
S16	00078	4419	393	7	5	*Clostridium*	62
S16	00079	4391	627	7	3	*Clostridium*	65
S16	00080	4387	413	6	4	*Clostridiales*	61
S16	00085	4283	438	7	5	*Fusobacterium*	65
S16	00087	4219	459	6	5	*Oribacterium*	73
S16	00088	4210	387	7	3	*Clostridium*	60
S16	00089	4208	285	7	3	*Clostridium*	58
S16	00091	4153	3549	5	2	*Parabacteroides*	49
S16	00093	4134	356	4	4	*Clostridium*	64
S16	00094	4105	502	8	4	*Lachnospiraceae*	54
S16	00096	4027	350	8	2	*Clostridium*	61
S16	00097	4030	415	6	3	*Clostridium*	66

S16	00098	4010	370	5	4	*Clostridium*	70
S18	00001	5009	10848	4	3	*Streptococcus*	75
S19	00001	4832	1060	8	4	*Prevotella*	84
S19	00004	4151	1045	5	2	*Streptococcus*	64

[a] Only ORFs encoding proteins of at least 100 aa are considered

[b] Number of translated ORFs with protein similarity to the organism in the closest relative column, as predicted by blastx
